# Ostrich egg shell as an accurate retrospective dosimeter using electron paramagnetic resonance technique

**DOI:** 10.1038/s41598-026-45071-6

**Published:** 2026-04-12

**Authors:** E. Aboelezz, M. A. Sharaf

**Affiliations:** Ionizing Radiation Metrology Department, National Institute of Standards (NIS), Giza, Egypt

**Keywords:** Electron paramagnetic resonance, Retrospective dosimetry, Ostrich egg shell, High dose, Energy dependence, Materials science, Physics

## Abstract

This study investigates the dosimetric features of Ostrich egg shell (Oesh) using Electron Paramagnetic Resonance (EPR) spectroscopy to evaluate its potential as an accurate retrospective dosimeter. Key dosimetric characteristics, including sensitivity, dose-response, UV light effect, and temporal and thermal stability, were systematically evaluated. The EPR spectra of γ-irradiated samples revealed six radiation-induced signals (S₁–S₆) corresponding to biocarbonate radicals (CO₃⁻, CO₃³⁻, and CO₂⁻), with the dosimetric peak centered at g = 2.0040 ± 0.0016. The material exhibits a highly linear dose-response relationship in the range of 0.3 Gy to 1 kGy (*R* > 0.999). With a detection limit of 0.21 Gy, Oesh demonstrates significantly higher sensitivity than other egg shell dosimeters. The EPR signal remained stable after seven days of storage at ambient temperature with less than 18% fading depending on modulation amplitude value. Crucially, exposure to UVA and UVC light did not affect the γ-induced radicals, confirming the robustness of the dosimetric centers. The ratio of the dosimetric peaks S_4_ to (S_4_ + S_5_) exhibits a time-dependent decay that can be modeled to determine the elapsed time since irradiation. Furthermore, the measured energy dependence factor for Cs-137 (662 keV) is 1.01 relative to Co-60 (1250 keV), confirming the material’s reliability across relevant photon energies greater than 100 keV. These results establish Ostrich egg shell as a promising, highly sensitive, and stable material for both retrospective dosimetry and general radiation monitoring applications.

## Introduction

The study of retrospective dosimetry has attracted great interest due to its potential uses in measuring radiation exposure in diverse situations, including medical, occupational, and accidental contexts. Finding the absorbed radiation dose after exposure requires retrospective dosimetry, particularly when conventional dosimeters were unavailable. Recent advancements in retrospective dosimetry materials have been made, particularly in the development of novel materials. Specifically, Aboelezz and Pogue^1^ introduced the dosimetric properties of newly synthesized nanomaterials and hybrid composites suitable for ionizing radiation dosimetry using techniques such as Electron Paramagnetic Resonance (EPR), Thermoluminescence (TL), and Optically Stimulated Luminescence (OSL). In the field of retrospective dosimetry, the search for new materials has been fruitful. A notable contribution is the updated comprehensive review by Yang et al.^2^, which summarizes materials suitable for this purpose. Among the various options, the use of biological materials such as avian egg shells has emerged as a particularly promising approach. Because of their structural stability, simplicity of collection, ease of use, and well-characterized radiation-sensitive features, these materials provide a non-invasive and reliable method to estimate radiation doses. In particular, Ostrich egg shells have gained attention as a possible material for retrospective dosimetry, because of their unique composition and structure. The egg shell, mainly composed of calcium carbonate about (94%), is a durable and naturally occurring material that has shown response to ionizing radiation. The remaining material consists of smaller amounts of organic matter (4%), magnesium carbonate (1%), and calcium phosphate (1%)^3^.

EPR spectroscopy is commonly regarded as an effective method for identifying radiation-induced defects in a variety of biominerals, including tooth enamel^4,5^ and egg shells^6–9^. EPR spectroscopy identifies unpaired electrons trapped in radiation-induced defects, yielding quantitative data on the dose absorbed by the material. This approach has been used for retrospective dosimetry on a broad variety of biological and inorganic materials, providing benefits such as high sensitivity, accuracy, and the ability to analyze samples with minimal pretreatment.

Previous studies have demonstrated that a linear relationship between the EPR signal of egg shells intensity and absorbed dose, with linearity extending up to at least 1 kGy and in some cases, up to 10 kGy. The minimum detectable dose has been reported to be as low as ~ 1 Gy, making egg shells a sensitive material for dosimetry^10^. Additional studies have explored the use of other avian egg shells, such as chicken egg shells^6,11^, for retrospective dosimetry using EPR, but the application of Ostrich egg shells has not been investigated enough^12^. Ostrich egg shells are very widely found at archaeological sites and are utilized for phylogenetic analysis^13^, reconstructions of social networks^14^, and paleoenvironmental studies^15^. Ostrich egg shell differs from those of smaller birds in terms of their physical properties, such as thickness, weight% of egg shell, mineral composition, and the presence of unique crystalline structures^16^. Particularly, it has the highest effective Young’s modulus among avian species (about 48 GPa)^17^. From well-defined nanoscale structures to accurate calcite crystallographic orientation, the biomineralization process is tightly controlled, resulting in a biomineral that cannot be found in other bird egg shells. This would explain why these egg shells are thick and have excellent mechanical qualities, especially when it comes to breaking, making them perfect models for bio-inspired materials and helping to preserve fossil material^18^. These differences may impact the EPR signal, potentially offering new avenues for dosimetric applications. However, Bhatti et al. (2013)^8^ investigated only the radiation dose response of various poultry eggs in the sterilization dose range (1–3 kGy); no publications have reported the distinctive EPR dosimetric features of Oesh. Given these factors, the potential of Ostrich egg shells as a reliable material for retrospective dosimetry using EPR spectroscopy is substantial. However, there remains a need for further research to explore the full range of EPR dosimetric features of Oesh, including their response to different radiation types, dose rates, and exposure conditions. In retrospective dosimetry, further detailed research is required to fully understand the impact of UV light on the EPR signal. Specifically, studies should focus on how UV exposure introduces an additional spectral component, known as the light-induced signal (LIS), which subsequently interferes with the reliable determination of the radiation-induced signal (RIS) component, compromising the accuracy of dose measurements^19,20^.

This study investigates the gamma radiation dosimetric characteristics of Ostrich egg shell (Oesh) using EPR spectroscopy. In particular, we evaluate their sensitivity, dose-response linearity, and dose-rate dependence, alongside the effects of UV light exposure. Furthermore, the thermal and temporal stability of the EPR signal is analyzed to assess its suitability for radiation monitoring and retrospective dosimetry.

## Materials and methods

### Sample preparation and irradiation

Ostrich egg shells with a thickness of 1.65 mm were purchased from a local market. The shells were cleaned and sterilized by immersion in absolute alcohol for 15 min, after which the inner soft membranes were manually removed. Following air-drying at room temperature, the Oesh pieces were crushed into a fine powder using a mortar and pestle. To investigate the influence of grain size, the powder was sieved into three distinct fractions using a standard mesh set: less than 106 μm, 106–212 μm, and larger than 212 μm.

Samples from the optimal grain size range were exposed to various air kerma levels of gamma radiation. Low and intermediate doses were delivered using a Theratron 780E (Nordion, Canada) at the National Institute of Standards (NIS). High-level doses were administered using a ^60^Co gamma source (Indian Gamma Chamber 4000 A) at the National Center for Radiation Research and Technology (NCRRT). For energy dependence studies, the samples were irradiated using ^60^Co, ^137^Cs, and 100 kV X-rays; the specific irradiation conditions for each beam quality are summarized in Table 1. Samples were irradiated under charged particle equilibrium (CPE) conditions using PMMA build up phantoms for ^137^Cs and ^60^Co. For the 100 kV X-ray source, however, a simple paper holder was used instead of PMMA to maintain the integrity of the energy spectrum and avoid excessive attenuation of the low-energy photons.

The kerma rates for the Theratron machine were calibrated using the NIS secondary standard system (0.6 cc PTW chamber and UNIDOS Webline electrometer), while high-dose calibrations were performed using the NIS alanine dosimetry system. Kerma rates for the ^137^Cs and 100 kV X-ray sources were assessed using PTW 1000 cc spherical and PTW 0.3 cc semiflex chambers, respectively. For each dose point, five aliquots were prepared and irradiated. Following exposure, samples were stored in the dark within sealed cuvettes. During storage and subsequent EPR readout, the relative humidity was strictly maintained between 40% and 60%.

### UV radiation

To investigate the effect of non-ionizing radiation on the EPR sensitivity of Oesh, UV radiation types A (UVA) and C (UVC) were used, with irradiance values of 0.72 and 2.7 mW/cm^2^, respectively. Two groups of samples, unirradiated and γ-irradiated Oesh, were exposed to UV radiation for 2 hours using a mercury ballasted tungsten filament (MBTF) lamp. Following exposure, the samples were stored in closed cuvettes in the dark. The UVA source was a GE Black Light F20-BLB (20 W, USA), while the UVC source was a GE Germicidal G13 T8 (20 W, Japan).

**Table 1:** Summary of irradiation conditions, dose rates and ranges for used beam qualities.

Beam quality	Dose range	Dose rate	Irradiation conditions
^137^Cs	2–10 Gy	0.15 Gy/min	Samples, with thickness of ≤ 3 mm, were irradiated in a unit of air kerma within a two-layers sandwich of 3 mm thickness of polymethylmethacrylate (PMMA).
100 kV X-rays	2–10 Gy	0.23 Gy/min.	Samples, with thickness of ≤ 2 mm, were irradiated inside a small sheet of paper (0.1 mm thickness).
^60^Co	0.3–2 Gy	0.11 Gy/min	Samples, with thickness of ≤ 4 mm, were irradiated in a unit of air kerma within a two-layers sandwich of 5 mm thickness of polymethylmethacrylate (PMMA).
	2–100 Gy	1.04 Gy/min	
	0.1–50 kGy	100 Gy/min	

### EPR measurements

For low and intermediate dose groups, the measurements of EPR spectra were performed within one hour after irradiation, while measurements of the high doses group were done after 7 days. EPR measurements were carried out at room temperature using an X-band EPR spectrometer (Bruker EMX) equipped with a high-sensitivity standard cylindrical resonator (ER4119HS) operating at 9.7 GHz and a modulation frequency of 100 kHz. A response time constant of 10 ms and a sweep time of 42 s were applied. The EPR operating parameters, including the microwave power and modulation amplitude, were optimized for dosimetric measurements. The optimum mass of Oesh powder was approximately 175 ± 10 mg, corresponding to a sample height of about 10 mm inside the tube.

### Energy dependence factor

The energy dependence factor for photons is defined as the ratio of dosimeter readout (EPR intensity of Ostrich egg shell) per dose, in terms of absorbed dose to air here, in a given beam energy relative to that for any reference beam quality, usually Co-60 γ-rays and expressed as^21,22^:

1 $${F}_{E,Co}=\frac{{\left(r/{D}_{air}\right)}_{E}}{{\left(r/{D}_{air}\right)}_{Co}}=\frac{{S}_{E}}{{S}_{Co}}=\frac{{\left[{\left(\stackrel{-}{{\mu}_{en}/\rho}\right)}_{air}^{dosim}\right]}_{E}}{{\left[{\left(\stackrel{-}{{\mu}_{en}/\rho}\right)}_{air}^{dosim}\right]}_{Co}}$$

where $\left(r/{D}_{air}\right)$ is the dosimeter readout $r$ per dose to air ${D}_{air}$which is calculated from the slopes of user beam quality $\left({S}_{E}\right)$ and reference beam quality $\left({S}_{Co}\right)$. [${\left(\stackrel{-}{{\mu}_{en}/\rho}\right)}_{air}^{dosim}$] is the ratio of mass energy absorption coefficient of the dosimeter, herein Oesh, to that of air averaged over the photon spectrum. The rightmost expression in Eq. (1) is used to calculate the energy factor theoretically from the NIST database^23^. The energies under investigation are 100 kV x-ray with effective energy of 40 keV, 662 keV for Cs-137, and 1250 keV for Co-60.

The effective x-ray energy was determined by achieving the half value layer (HVL) complied with recommended values by the Consultative Committee for Ionizing Radiation (CCRI) beam quality. The mass energy absorption coefficient (${\mu}_{en}/\rho$) for Oesh was calculated based on its mineral composition, which constitutes 97% of the total shell mass. Using the following equation^22^, the mineral fraction was analyzed using the distribution reported in^24^[CaCO_3_ (97.4%), Mg_3_(PO_4_)_2_ (1.9%), Ca_3_(PO_4_)_2_ (0.7%)]:

2 $${\left(\raisebox{1ex}{${\mu}_{en}$}\!\left/\!\raisebox{-1ex}{$\rho$}\right.\right)}_{compound}=\sum_{i=1}^{n}{W}_{{Z}_{i}}{\left(\raisebox{1ex}{${\mu}_{en}$}\!\left/\!\raisebox{-1ex}{$\rho$}\right.\right)}_{{Z}_{i}}$$

where $\left({W}_{{Z}_{i}}\right)$ is the weight fraction of the element to the total molecular weight for the (i) element with atomic number (Z), and calculated from the following equation:

3 $${W}_{{Z}_{i}}=\raisebox{1ex}{$\left({n}_{i}*{Z}_{i}\right)$}\!\left/\!\raisebox{-1ex}{$\sum_{i=1}^{n}\left({n}_{i}*{Z}_{i}\right)$}\right.$$

where (${n}_{i}$) is the number of element atoms per molecule.

To estimate the combined standard uncertainty of the relationship slope of the dose response in all qualities $\left({S}_{E}\right)$relative to that of ^60^Co $\left({S}_{Co}\right)$, the propagation law of uncertainties was applied as follows^22,25^:

4 $$u\left(\frac{{S}_{E}}{{S}_{Co}}\right)=\sqrt{{u}^{2}\left(\frac{{S}_{E}}{{S}_{Co}}\right)}=\sqrt{\frac{1}{{\left({S}_{Co}\right)}^{2}}{u}^{2}\left({S}_{E}\right)+\frac{{\left({-S}_{E}\right)}^{2}}{{\left({S}_{Co}\right)}^{4}}{u}^{2}\left({S}_{Co}\right)+2\frac{\left(-{S}_{E}\right)}{{\left({S}_{Co}\right)}^{3}}u\left({S}_{E}\right)u\left({S}_{Co}\right)r\left({S}_{E},{S}_{Co}\right)}$$

where ${u}^{2}\left({S}_{E}\right)$ and ${u}^{2}\left({S}_{Co}\right)$ are the variance; $u\left({S}_{E}\right)$and $u\left({S}_{Co}\right)$ are the combined standard uncertainty of the relationship slope of the dose response in all qualities and ^60^Co, respectively. $r\left({S}_{E},{S}_{Co}\right)$ represents the correlation coefficient between the two respective slopes, calculated for the same number of measurements.

## Results and discussions

### EPR spectrum characteristics

#### EPR spectrum shape

**Fig. 1:**
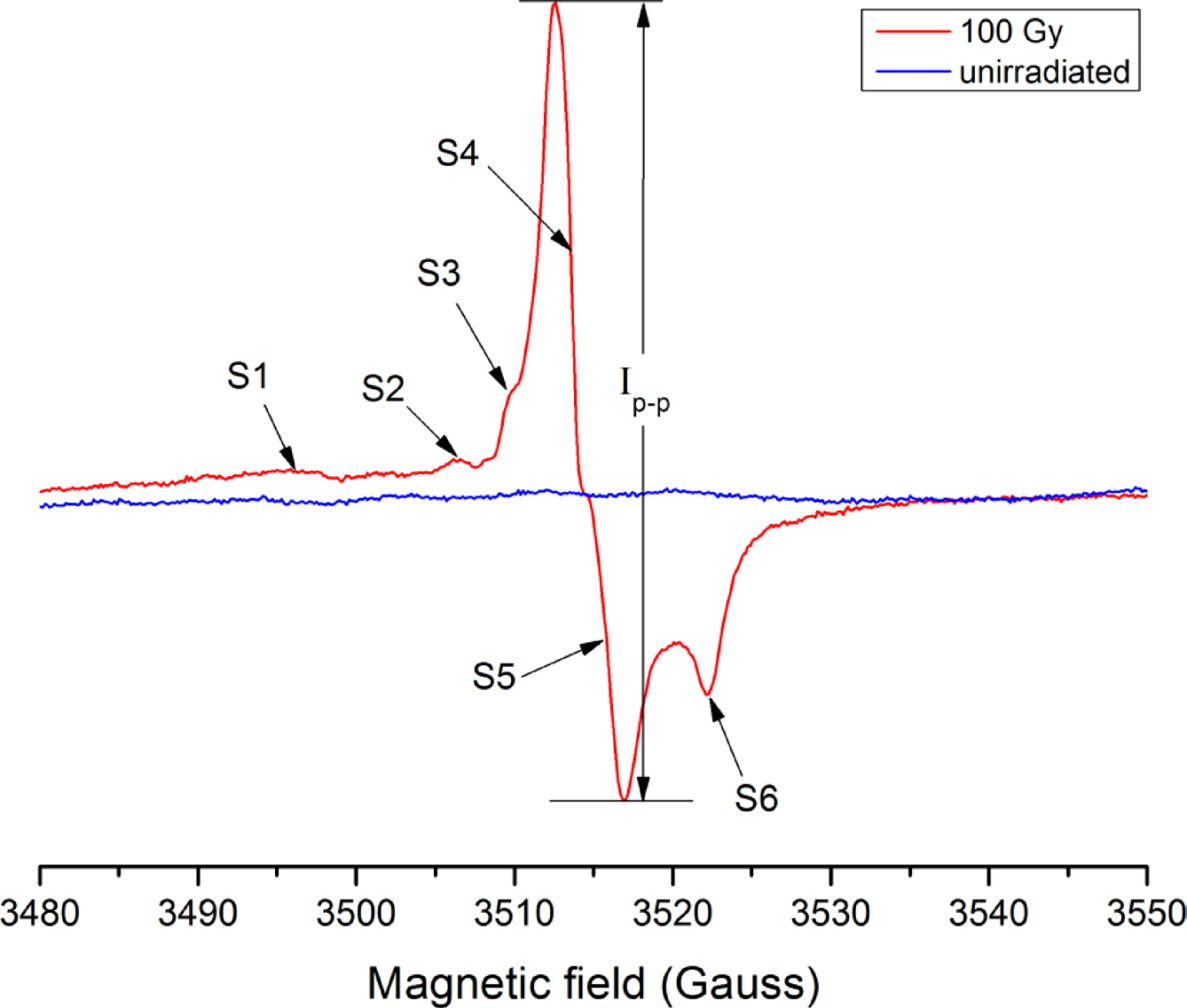
Spectra of the Ostrich egg shell for unirradiated and 100 Gy of γ-rays measured immediately after irradiation and at 1 G modulation amplitude and 20 mW microwave power.

Figure 1 shows EPR spectra of the Ostrich egg shell sample, comparing an irradiated sample (100 Gy, red line) with an unirradiated one (blue line). The spectra are plotted as a function of magnetic field (in Gauss) along the x-axis, with the signal intensity along the y-axis. The unirradiated sample exhibits a nearly flat baseline with minimal signal, indicating the absence or very low concentration of paramagnetic centers. In contrast, the irradiated sample displays distinct radiation-induced apparent signals labeled S_1_ through S_6_ with g-factors in Table 2, indicating the formation of radiation-induced free radicals or paramagnetic defects as a result of gamma irradiation. The dosimetric signal intensity was quantified using the peak-to-peak amplitude (Iₚ₋ₚ) of the composite EPR spectrum. The magnetic field separation between the maximum intensity value of S_4_ and minimum value of S_5_ at g-factor of 2.0040 ± 0.0016 is approximately 5 G. This separation reflects the overlap of multiple carbonate-related radical components rather than the intrinsic linewidth of a single paramagnetic species. The additional peaks (S_1_–S_6_) suggest a complex radical environment, possibly due to different radical species or varying local environments within the calcite matrix of the egg shell. Based on the similar fundamental composition of all avian egg shells, their RIS is expected to be nearly the same^7,8^, even though the strength of their response to radiation (response intensities) may differ. This consistency means we can compare the origins of the radicals observed in Oesh with results from any avian egg shell studied previously. We conclude that the Ostrich egg shell’s spectrum is made up of the orthorhombic forms of the molecular radicals: CO_3_^−^, CO_3_^3−^, and CO_2_^−^.

**Table 2:** The obtained values of g-factors for radiation induced signals (RIS) in EPR spectrum of the Oesh.

Signal	g-factor	Radical
S_1_	2.0148 ± 0.0015	CO_3_^−^^7,26^
S_2_	2.0087 ± 0.0014	CO_3_^−^^7,26^
S_3_	2.0067 ± 0.0014	CO_3_^−^^7,26^
S_4_	2.0046 ± 0.0015	CO_3_^3−^^9,26^
S_5_	2.0033 ± 0.0014	CO_2_^−^^9,26^
S_6_	1.9998 ± 0.0015	CO_2_^−^^9,26^

#### Microwave power

**Fig. 2:**
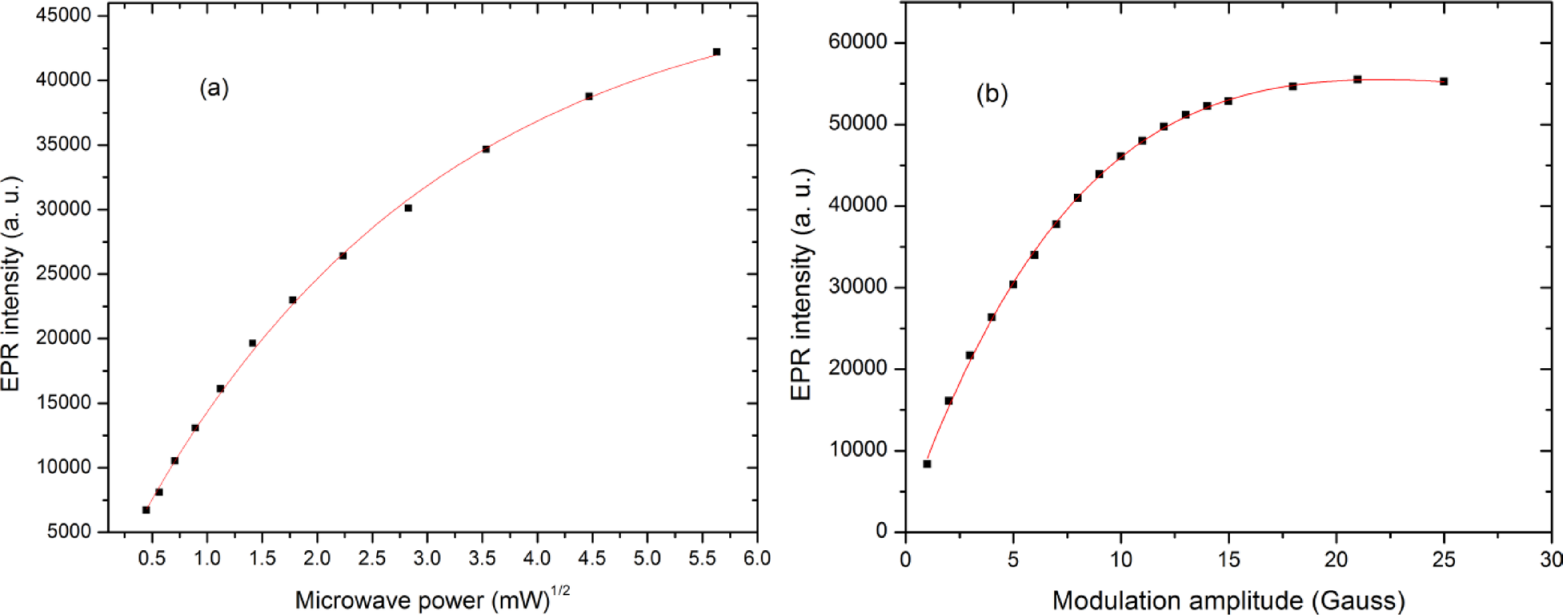
EPR spectrum properties including the effect of both the variation of microwave power (**a**) and modulation amplitude (**b**) on EPR intensity of an Ostrich egg shell irradiated to 100 Gy.

Microwave power should be carefully adjusted to avoid settings that are excessively high, which can lead to signal saturation, or excessively low, resulting in a diminished Signal-to-Noise ratio (S/N)^27^. Figure 2a shows the dependence of the EPR peak-to-peak intensity on the square root of microwave power for an Oesh sample irradiated to 100 Gy with a Co-60 source. At low microwave powers up to 1.25 mW, the signal intensity increases nearly linearly with the square root of power, reflecting efficient absorption and minimal saturation of the paramagnetic centers. As the microwave power increases further, the growth in signal intensity begins to deviate from linearity, exhibiting an exponential behavior without reaching full saturation. The saturation occurs when the spin relaxation processes are no longer fast enough to keep up in the higher energy level with the increasing microwave field, leading to reduced efficiency in signal enhancement. This trend underscores the critical importance of optimizing microwave power during EPR measurements. For irradiated Oesh to 100 Gy of gamma rays, the curve suggests an optimal operating region before the saturation plateau, ensuring maximum sensitivity without compromising spectral quality. This behavior is typical for radiation-induced radicals in carbonate-based biominerals^5,28^, confirming that the optimum range lies between 10 and 32 mW. Since it does not reach the saturation, the power of 20 mW was chosen for the measurements in this paper.

#### Modulation amplitude

The effect of modulation amplitude on the EPR signal intensity (I_p-p_) of an irradiated Ostrich egg shell is displayed in Fig. 2b. As the modulation amplitude increases from a very low value, the signal intensity also increases, leading to a better S/N ratio. However, once the modulation amplitude exceeds a certain value, typically on the order of the signal linewidth, the spectral lines begin to broaden and distort. For accurate dosimetry, it is critical to select an optimal modulation amplitude that provides a good S/N ratio without causing significant line broadening. This optimal range was experimentally determined to be 3–5 Gauss (G). The most intense signal, corresponding to a radiation-induced carbonate radical, is used for dose assessment, and this figure illustrates the importance of proper instrument settings to ensure the accuracy of the final dose estimation.

### Grain size effect

Angular dependence of EPR signals in avian egg shells has been previously reported^10^, often resulting in poor reproducibility and variability in EPR intensity with changes in sample orientation. The use of powdered samples with reduced grain size mitigates this effect by promoting random orientation of crystallites, thereby averaging anisotropic contributions and stabilizing the measured EPR signal intensity^29,30^. In this study, the effect of grain size on EPR intensity and measurement variability of a 100 Gy irradiated Oesh was investigated across three size ranges: >212 μm, 106–212 μm, and < 106 μm. As expected, the standard deviation percentage (SD%) of five repeated measurements taken immediately after irradiation decreased with decreasing grain size (2.6%, 1%, and 0.6% for > 212 μm, 106–212 μm, and < 106 μm, respectively). This improvement in reproducibility is attributed primarily to enhanced orientation averaging and improved packing homogeneity for finer powders.

In addition to improved reproducibility, a modest increase in EPR signal intensity (≈ 2%) was observed for the two smaller grain size fractions compared to the > 212 μm fraction, while the intensities of the 106–212 μm and < 106 μm fractions were nearly identical (SD% ≈ 0.01%). This enhancement in sensitivity for finer grains can be attributed to several concurrent physical effects. First, the increased surface-to-volume ratio in smaller particles facilitates more efficient microwave penetration and reduces field inhomogeneities within individual grains, leading to more effective excitation of paramagnetic centers. Second, reduction in grain size may influence spin–lattice relaxation processes by modifying phonon spectra and defect environments, thereby improving saturation behavior under fixed microwave power conditions^31^. Third, mechanical grinding can introduce microstructural strain and lattice imperfections, which may act as additional trapping or stabilization sites for radiation-induced carbonate radicals, contributing to a slight increase in detectable EPR signal. The absence of a significant difference between the two smallest grain size fractions suggests that these effects reach a practical saturation once sufficient powder fineness is achieved, beyond which further size reduction does not yield additional sensitivity gains. The deviation of the present results from earlier observations reported for chicken egg shell^10^ is therefore not attributed to intrinsic material hardness alone, but rather to a combination of particle size distribution, microstructural characteristics, and measurement conditions. Based on the combined considerations of signal intensity, reproducibility, and practical sample handling, the grain size range of 106–212 μm was selected for all subsequent EPR measurements in this study.

### Fading effect

**Fig. 3:**
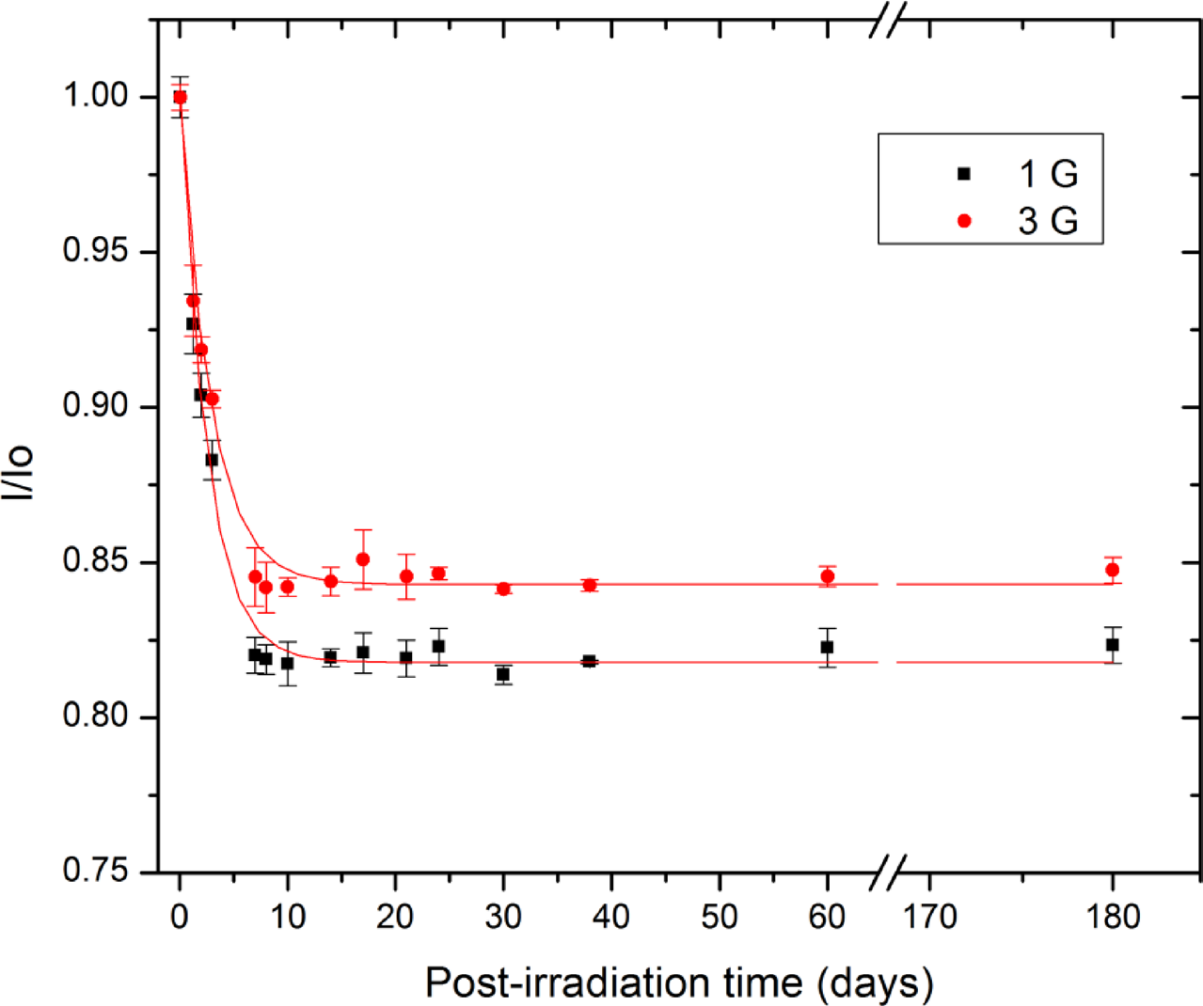
The fading effect on the EPR intensity of exposed Ostrich egg shell to 100 Gy. The exponential fitting was applied for both modulation amplitude; 1 G (black square) and 3 G (red circle). The error bars represent the standard deviation of five aliquots.

The post-irradiation stability of radiation-induced paramagnetic centers in Oesh was evaluated by monitoring the EPR signal fading of samples irradiated to 100 Gy and stored at ambient laboratory conditions for up to six months (Fig. 3). To assess the influence of measurement parameters on the observed fading behavior, EPR measurements were performed using two modulation amplitudes, 1 G and 3 G. The EPR intensities were normalized to their respective initial values measured immediately after irradiation and plotted as a function of post-irradiation time. For both modulation amplitudes, the dosimetric EPR signal exhibits an initial decrease during the first several days after irradiation, followed by a pronounced stabilization phase extending over the remainder of the observation period. The total fading observed prior to stabilization amounts to approximately 18% for measurements performed at 1 G and 15% at 3 G. This behavior can be adequately described by a first-order decay of a fast component (Eq. 5) superimposed on a dominant stable fraction (${A}_{\mathrm{s}\mathrm{t}\mathrm{a}\mathrm{b}\mathrm{l}\mathrm{e}}$), reflecting the coexistence of short-lived and long-lived radiation induced radical species in Oesh.

5 $$\frac{I\left(t\right)}{{I}_{0}}={A}_{\mathrm{f}\mathrm{a}\mathrm{s}\mathrm{t}}{*e}^{-t/{\tau}_{\mathrm{f}\mathrm{a}\mathrm{s}\mathrm{t}}}+{A}_{\mathrm{s}\mathrm{t}\mathrm{a}\mathrm{b}\mathrm{l}\mathrm{e}}$$

The fitted decay constants of the fast component (${\tau}_{\mathrm{f}\mathrm{a}\mathrm{s}\mathrm{t}}$) were determined to be 2.38 days and 2.85 days for modulation amplitudes of 1 G and 3 G, respectively, while the corresponding fast-decaying fractions (${A}_{\mathrm{f}\mathrm{a}\mathrm{s}\mathrm{t}}$) were 0.184 and 0.157. These values indicate that the intrinsic post-irradiation radical decay kinetics are only weakly influenced by the modulation amplitude, confirming that the observed differences primarily arise from spectral averaging and resolution effects rather than from fundamental changes in radical stability.

The stabilization of the EPR signal after approximately seven days suggests that reliable dose readout should be performed either immediately (within 2 h) after irradiation or after this stabilization period, once transient radical contributions have decayed. Beyond this time, the remaining signal is dominated by highly stable carbonate radicals, which are known to persist for long durations in calcite-based biominerals and form the basis of long-term retrospective dosimetry.

**Fig. 4:**
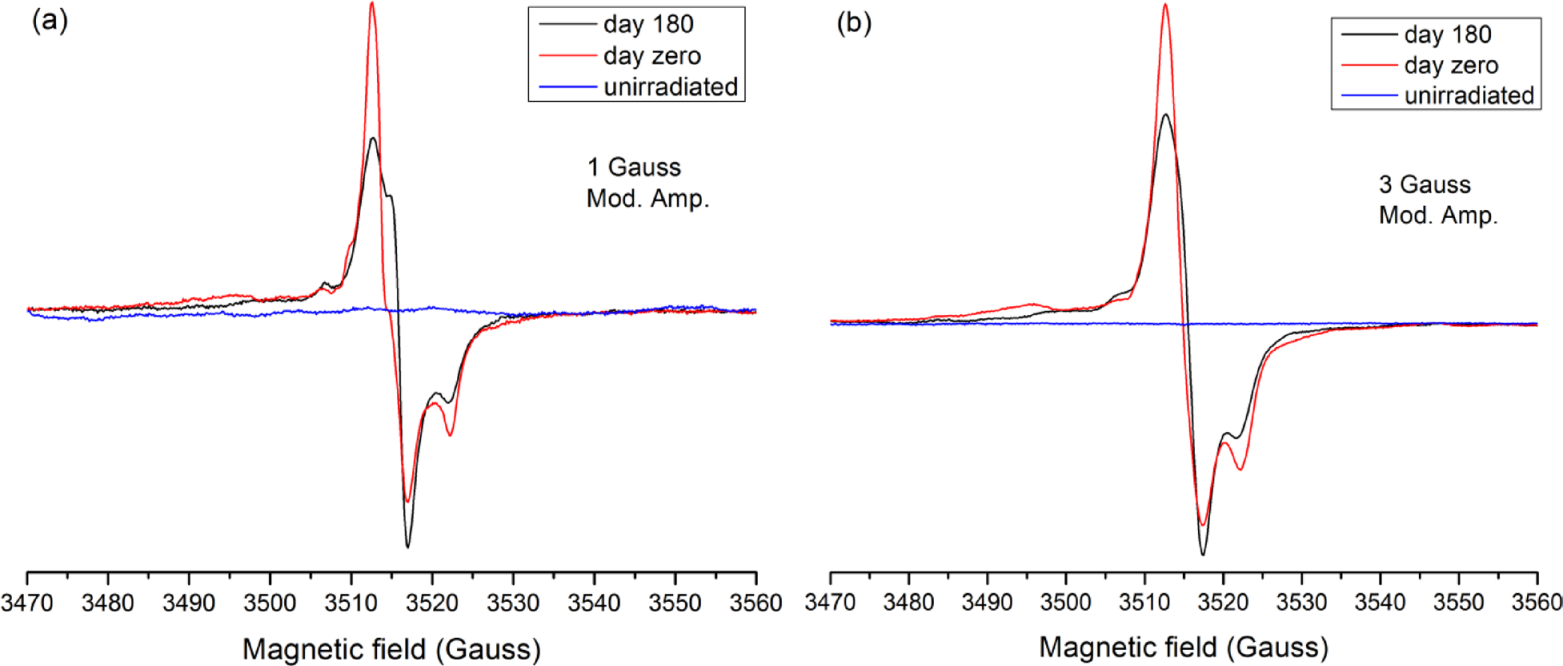
Fading effect on the EPR spectra of irradiated Ostrich egg shell to 100 Gy for two different modulation amplitudes: 1 Gauss (**a**) and 3 Gauss (**b**). Blue spectrum for unirradiated samples, red spectrum for immediately read out after irradiation, and black spectrum for the read out of 180 days after irradiation.

Representative EPR spectra recorded immediately after irradiation and after prolonged storage further illustrate the fading behavior as shown in Fig. 4a and b. The decrease in overall signal intensity over time reflects the decay of unstable radical species. Notably, Fig. 4a reveals shifts in the relative intensity of specific apparent EPR spectral features, as certain components become more prominent while others diminish. However, due to the strong overlap of multiple carbonate-related radical signals in first derivative EPR spectra, such apparent changes should be interpreted with caution. A decrease in one overlapping component may lead to an apparent enhancement of another, even in the absence of a genuine increase in radical concentration. Without spectral decomposition or simulation of the individual components, quantitative conclusions regarding the evolution of specific radical species cannot be drawn. Additionally, the observed difference in fading between 1 and 3 G modulation can be due to stronger averaging of various EPR lines in the modulation broadened 3G spectra. In general, radiation-induced radicals in calcite-based biominerals are known to include both short-lived transient species and more persistent carbonate radicals, such as CO₂⁻, which dominate the EPR spectrum at longer storage times^32,33^.

**Fig. 5:**
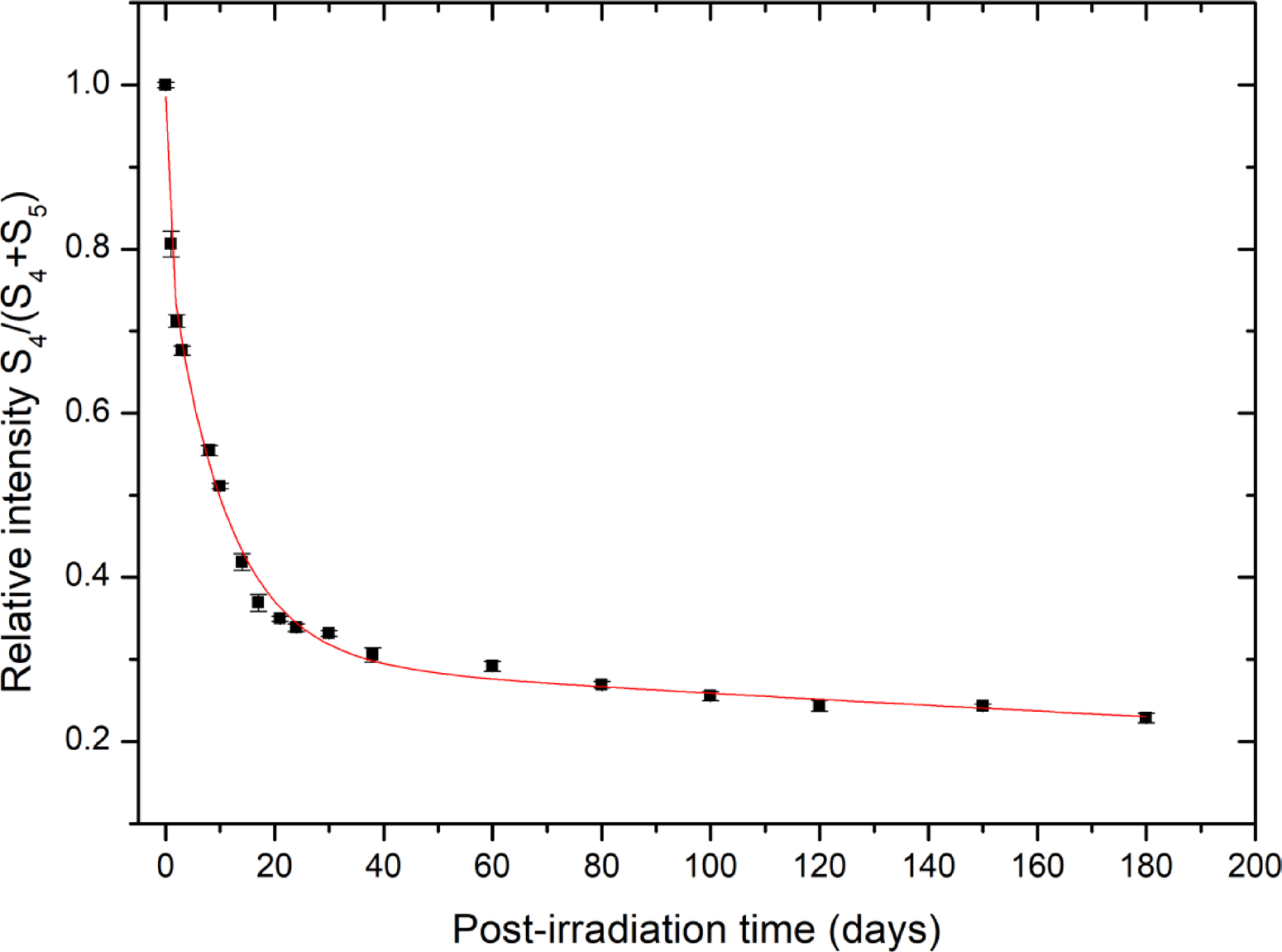
The relation between the relative intensity of S_4_ to (S_4_ + S_5_) for Oesh irradiated to 50 Gy and post-irradiation time over 6 months.

The time dependence of the apparent S_4_/(S_4_ + S_5_) intensity ratio is presented in Fig. 5. The ratio decreases monotonically with post-irradiation time, reflecting the differential fading rates of overlapping spectral contributions within the composite EPR signal. The experimental data can be empirically described by a third-order exponential function (R^2^ = 0.997, Eq. 6), providing a mathematical representation of the observed trend. It should be emphasized that this fitting function is used as a descriptive and calibration tool rather than as mechanistic evidence of direct conversion between distinct radical species. The evolution of the S_4_/(S_4_ + S_5_) ratio most likely arises from differences in kinetic stability among overlapping radical components contributing to the S_4_ and S_5_ extrema.

6 $$y=0.192{e}^{-t/0.572}+0.508{e}^{-t/10.8}+0.299{e}^{-t/688}$$

The monotonic and reproducible behavior of this ratio with time enables its use as a practical retrospective indicator of post-irradiation interval. By inverting the empirical fitting function, the elapsed time after irradiation can be estimated within the investigated temporal range. Thus, the S_4_/(S_4_ + S_5_) ratio provides a useful dosimetric parameter for temporal reconstruction without requiring full spectral deconvolution.

Overall, the fading behavior of Oesh is characterized by an initial transient phase followed by excellent long-term stability, supporting its suitability for retrospective EPR dosimetry. The consolidation of fading results highlights the dominant physical processes governing signal stability while avoiding over-interpretation of overlapping spectral features.

### Dose response

**Fig. 6:**
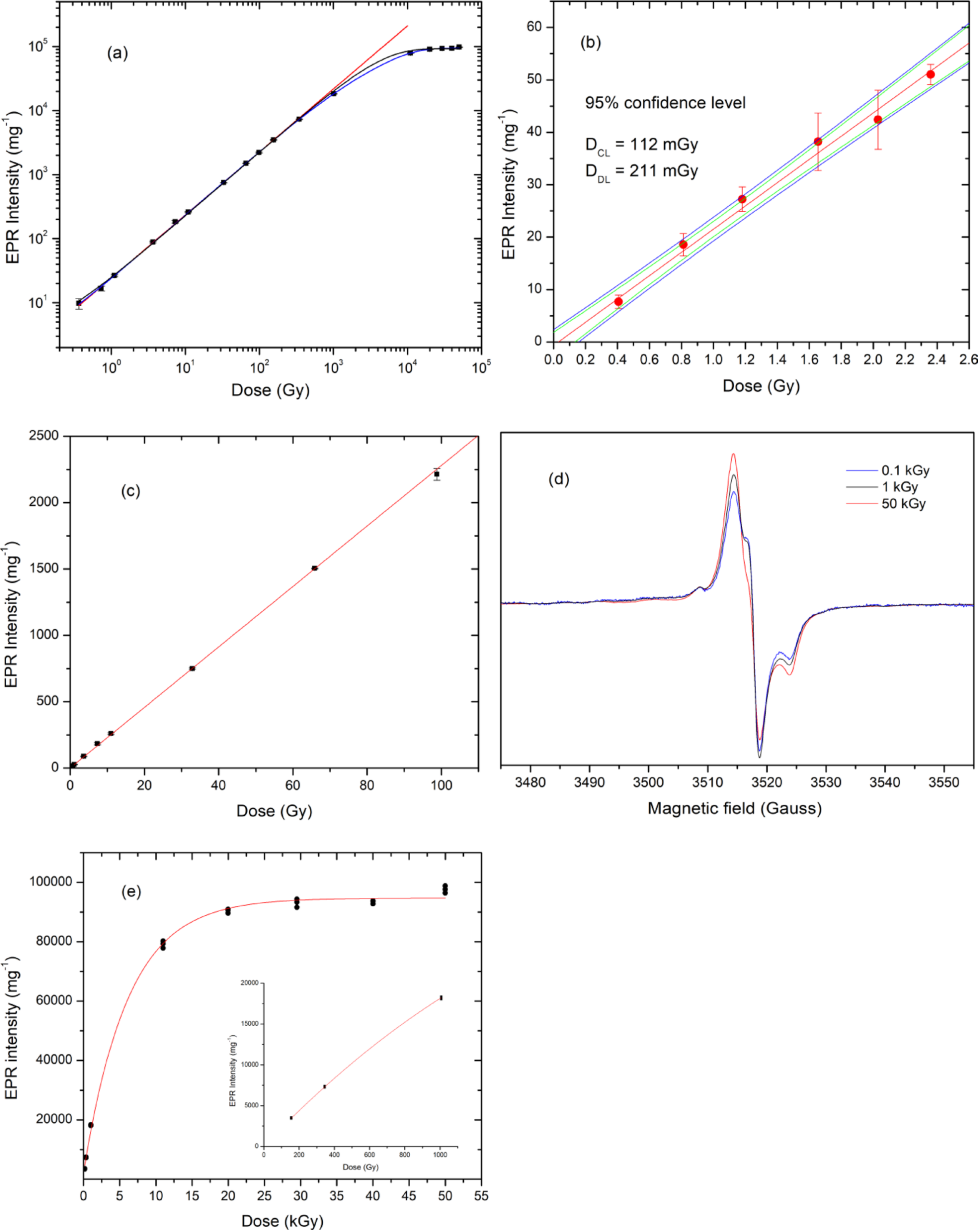
**a** Dose response behavior of Ostrich egg shell from 0.3 Gy to 50 kGy of γ-rays from ^60^Co-source measured after 7 days of irradiation and at 3 G modulation amplitude on the log-log scale. **b** Normalized EPR intensity as a function low radiation dose. Detection limit (D_DL_) and critical level (D_CL_) of Ostrich egg shell of gamma irradiated doses. **c** Dose response of intermediate range. **d** EPR spectrum for 0.1, 1 and 50 kGy of γ-rays from ^60^Co-source measured after 15 days of irradiation. **e** Dose response from 0.1 to 50 kGy, the inset shows dose response in the range from 0.1–1.1 kGy. Error bars at each dose point represent the standard deviation of I_p-p_ of five aliquots.

Figure 6a illustrates the dose response relationship of the Oesh EPR signal over a wide absorbed dose range from ^60^Co, extending from sub-gray levels up to 50 kGy, plotted on log-log graph. To ensure comparability across the full range and to minimize the influence of early-time fading, spectra used for evaluating global dose-response behavior were performed 7 days post-irradiation, when the signal is dominated by stable radical species. At low and intermediate doses (up to ~ 1 kGy), the EPR intensity increases nearly linearly (red line) with absorbed dose (slope = 0.988 ± 0.003, R^2^ = 0.9999), indicating efficient conversion of deposited energy into paramagnetic centers and negligible saturation of available trapping sites. This linearity across several orders of magnitude reflects the high sensitivity of Oesh in the low-dose region and confirms its suitability for retrospective dosimetry. With increasing dose, the response progressively deviates from linearity and approaches saturation at the highest doses. Such behavior is typical of solid-state dosimeters in which the number of radiation-sensitive precursor sites is finite and radical–radical interactions become increasingly important at high spin densities. Consequently, purely linear or polynomial descriptions lack physical significance over extended dose ranges.

To describe this non-linear behavior over the extended dose range, physically motivated exponential saturation models, as described in Appendix, were applied. These models are commonly used to describe solid state dosimeters in which the concentration of radiation-sensitive precursor sites is finite and progressively exhausted with increasing absorbed dose. The entire dose response dataset can be satisfactorily described by a single hit (first order) saturation model (A.1.1.). Fitting the full dose range (black line in Fig. 6a) using this model yields the equation:

7 $$I\left(D\right)=2.43+9.23*{10}^{4}\left[1-exp\left(\frac{-D}{4071}\right)\right]$$

with a high goodness of fit (R^2^ ≈ 0.999). The characteristic dose ${D}_{0}$ ≈ 4.1 kGy indicates that the dominant dosimetric component remains far from saturation up to several kilograys, explaining the extended quasi-linear response observed at intermediate doses. Although the single-hit model provides an excellent global fit, systematic deviations are observed at intermediate doses, suggesting the presence of additional kinetic contributions. While using two-component (multi-hit) saturation model (A.1.2.), the fitting of full dose range (blue line in Fig. 6a) yields the equation:

8 $$I\left(D\right)=1.03+8.62*{10}^{4}\left[1-exp\left(\frac{-D}{6374}\right)\right]+8*{10}^{3}\left[1-exp\left(\frac{-D}{825}\right)\right]$$

This model yields a superior correlation (R^2^ ≈ 0.9999) and reveals two characteristic saturation doses of about 0.83 kGy and about 6.4 kGy. The ${D}_{02}$​ component saturates rapidly and contributes mainly at low-to-intermediate doses, while the ${D}_{01}$​ component dominates the response at high doses. Importantly, these components should be regarded as apparent kinetic contributions rather than obvious evidence of distinct radical species, as spectral deconvolution was not performed.

#### Low dose response

Figure 6b illustrates the dependence of the EPR peak-to-peak heights, normalized to sample weight (mg), on absorbed gamma dose for Oesh measured immediately after irradiation. Net peak-to-peak heights were obtained by subtracting the signal of an unirradiated sample each measured one to minimize the instrumental noise contribution. A weighted linear least square fits on a linear-linear scale yields a slope of (22.18 ± 0.7), and an intercept of (–0.66 ± 0.9), with R-value of 0.998. The regression line (red line in Fig. 6b) represents the mean EPR intensity obtained from five samples at each gamma dose. The standard error percentage of the slope is approximately 3.2%, which is somewhat elevated compared to reference EPR dosimeters, such as alanine; however, this level of error remains acceptable and indicates a significant difference in performance. Following the recommendations of IUPAC (1995)^34^ and the ISO 11,929 series (2019)^35^, the critical level (D_CL_), detection limit (D_DL_), and quantification limit (D_QL_) were evaluated using OriginPro 8.5 software, as detailed by Aboelezz et al. (2015)^36^. The corresponding estimated values are 112, 211, and 1120 mGy, respectively. These results highlight a significant advancement in sensitivity, allowing for detection limits below one-fifth of the previously reported detection limit for chicken egg shells (1.2 Gy)^10^. The background variability contributing to detection limits is dominated from the intrinsic material heterogeneity rather than instrumental noise. Notably, the detection limit for Ostrich egg shells utilized in radiation dosimetry is remarkably low, underscoring its sensitivity and effectiveness as a retrospective EPR dosimeter.

#### Intermediate dose response

The relation between the EPR peak-to-peak heights normalized to weight (mg) of Oesh dosimetric signals measured immediately after irradiation is demonstrated in Fig. 6c, for the intermediate range of the absorbed doses (2–100 Gy). The slope of the regression line equation that was obtained from the weighted linear fitting of this relation on a linear-linear scale is (22.77 ± 0.08) and the intercept (2.43 ± 1.28), with R-value of 0.99996. The close agreement between the slopes obtained in the low- and intermediate-dose regions indicates stable dosimetric behavior across these ranges and confirms that fading effects are negligible on the time scale of immediate post-irradiation measurements.

#### High dose response

Figure 6d presents normalized EPR spectra measured 15 days after irradiation at doses of 0.1, 1, and 50 kGy. While the overall spectral shape and g-value remain unchanged, the relative intensities of spectral extrema vary systematically with dose. In particular, the peak-to-peak intensity ratio between the S_4_ and S_5_ extrema increases monotonically from approximately 0.19 at 0.1 kGy to 0.74 at 50 kGy. This behavior indicates differential saturation of overlapping spectral contributions rather than uniform scaling of a single component.

The high-dose response (0.1–50 kGy), shown in Fig. 6e, is well described by the first-order saturation function:

9 $$I\left(D\right)=2300+9.24*{10}^{5}\left[1-exp\left(\frac{-D}{6.12}\right)\right]$$

with R² ≈ 0.998, confirming saturation of the dominant dosimetric component at approximately 6 kGy, as obtained in Eq. 8. The inset highlights the lower dose range (100–1000 Gy), where the response remains nearly linear, corresponding to the early stage of trap occupancy prior to significant saturation effects.

Overall, the dose response fitting, spectral intensity ratio evolution, and fading behavior support a dual component dosimetric response in Ostrich egg shell. While the entire dataset can be adequately described by a single hit saturation model, the multicomponent approach provides additional insight into the presence of at least two apparent saturation processes with different characteristic doses. This behavior is primarily attributed to exhaustion of radiation-sensitive carbonate precursor sites, with additional contributions from enhanced radical–radical interactions at elevated spin densities, rather than unambiguous formation of distinct radical species.

### Energy dependence

The energy dependence factor of Oesh sample was determined from the slope ratio of dose response to different radiation qualities, normalized to that of the ^60^Co reference beam (Table 3). All doses reported in this study correspond to absorbed dose to air (air kerma), as realized by calibrated reference dosimetry systems at the sample position. Based on this approach, the energy dependence factors were calculated using Eq. (1) for different photon beams. The combined standard uncertainty of the slope ratio, comparing each beam quality $\left({S}_{E}\right)$to the reference ^60^Co beam $\left({S}_{Co}\right)$, was evaluated using Eq. (4). Table 3 also lists the energy dependence factors, both measured and calculated, and the combined uncertainty for the measured values.

**Fig. 7:**
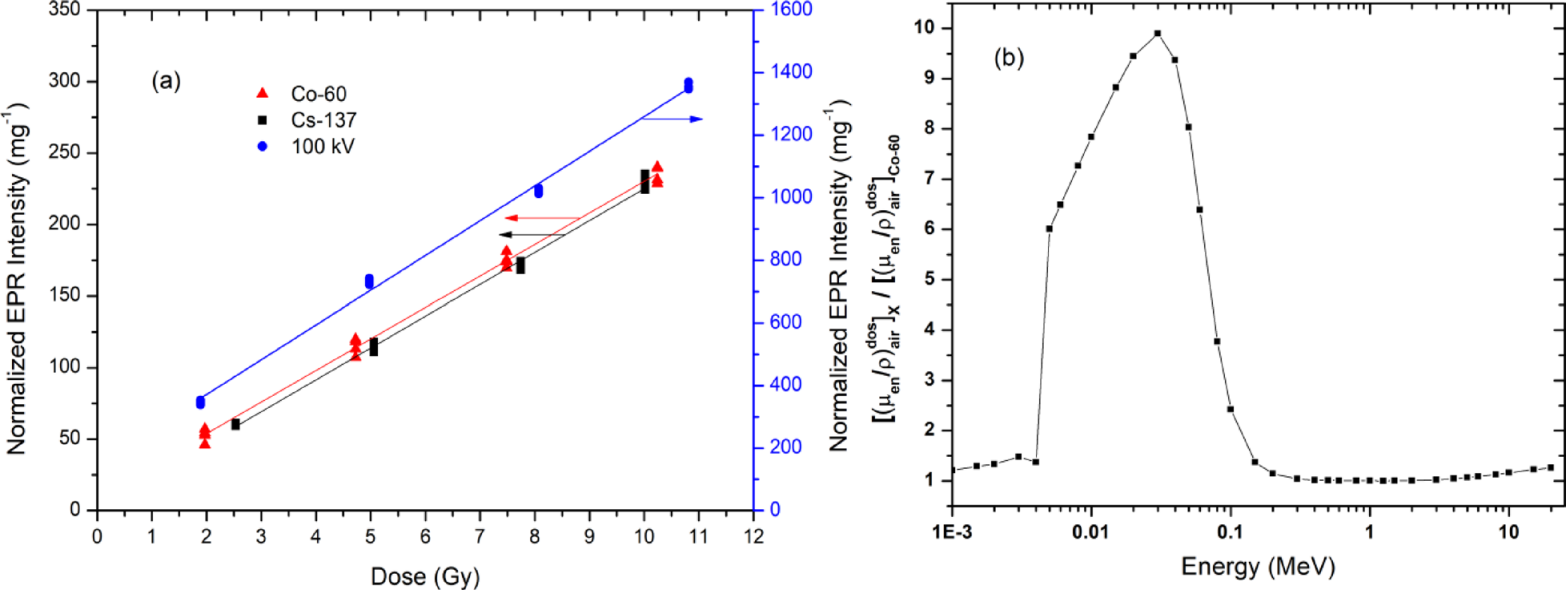
The dose response of Ostrich egg shell for different beam qualities **a** 100 kV, Cs-137 and Co-60 in a dose range of 1–10 Gy. The theoretical explanation is shown in **b** by the relation between relative mass energy absorption coefficients and effective photon energy.

The significant differences observed in the slopes of the dose–response curves (Fig. 7; Table 3) are primarily attributed to changes in photon beam energy. This trend is qualitatively consistent with expectations based on mass energy-absorption coefficient ratios between egg shell material and air. Such behavior is anticipated, as Oesh is not tissue-equivalent and exhibits a relatively high effective atomic number (Z_eff_​ = 14.9). As a consequence, an over-response relative to tissue and air is expected at lower photon energies, where photoelectric absorption becomes increasingly significant. It should be noted, however, that for powdered detectors the experimentally observed slope differences reflect the combined effects of photon interaction probabilities and secondary electron transport under the applied irradiation conditions, rather than mass energy-absorption coefficients alone.

**Table 3:** Summary of fitting results of EPR measurements of irradiated Oesh to a dose range of 1–10 Gy. In this fitting, a statistically weighted least-squares linear regression method was used. The measured factor, the associated combined standard uncertainty and the calculated factor for each beam quality relative to ^60^Co.

Nominal energy	Mean energy (keV)	Slope		Correlation coefficient	Measured ${\boldsymbol{F}}_{\boldsymbol{E},\boldsymbol{C}\boldsymbol{o}}$	u_c_	Calculated ${\boldsymbol{F}}_{\boldsymbol{E},\boldsymbol{C}\boldsymbol{o}}$^*^
		Value (Gy^− 1^. mg^− 1^)	S.E. %				
100 kV	40^**^	133.52	1.54	0.9971	6.06	18.6 × 10^− 3^	9.37
^137^Cs	662	22.26	1.66	0.9960	1.01	2.2 × 10^− 3^	1.01
^60^Co	1250	22.04	1.81	1	----	----	----

*Calculated factors were determined using the data listed in tables of mass energy absorption coefficients from NIST database^23^.

**The reported value represents the effective photon energy (not mean energy) derived from the measured HVL (0.149 mm Cu) using tabulated X-ray mass attenuation coefficients from the NIST database (https://physics.nist.gov/PhysRefData/XrayMassCoef/tab3.html).

The egg shell samples were irradiated as compact powder layers with thicknesses in the range of 2–4 mm and grain sizes of 106–212 μm. Under such conditions, the detector response is influenced by both photon interactions and secondary electron transport. When the dimensions of the sensitive volume are comparable to the range of secondary electrons, the absorbed dose in the detector material may deviate from the air kerma and is governed by an intermediate cavity behavior, as described by Burlin’s cavity theory^37^. Consequently, the absorbed dose ratio between egg shell and air is affected not only by mass energy-absorption coefficients but also by stopping-power considerations.

For Cs-137 and Co-60 irradiations, the high photon energies and the use of sufficiently thick surrounding material ensure CPE conditions. Under CPE, the contribution of secondary electron transport is largely balanced, and the experimentally determined energy dependence factors are expected to closely reflect the intrinsic material response. The good agreement observed between measured and calculated values of (${F}_{E,Cs}$) for ^137^Cs and ^60^Co supports this assumption and confirms that electron equilibrium effects are adequately satisfied for these radiation qualities^38^. In contrast, for the 100 kV X-ray beam, deviations between measured and calculated energy dependence factors are observed. These deviations are attributed primarily to limitations of the theoretical approximation rather than to experimental uncertainty. The calculated factor relies on an effective photon energy derived from half-value layer measurements, which represents a simplification of the inherently polyenergetic X-ray spectrum. The full spectral distribution for this beam quality was not available and was therefore not used. In addition, PMMA buildup was intentionally not applied for the 100 kV irradiations, since the use of buildup material would significantly harden the beam and alter the CCRI-defined beam quality, particularly by removing low-energy photons that contribute substantially to the detector response. As a result, the determined energy dependence factor inherently includes the combined effects of the actual photon spectrum and secondary electron transport under the applied irradiation conditions.

### UV radiation effect

**Fig. 8:**
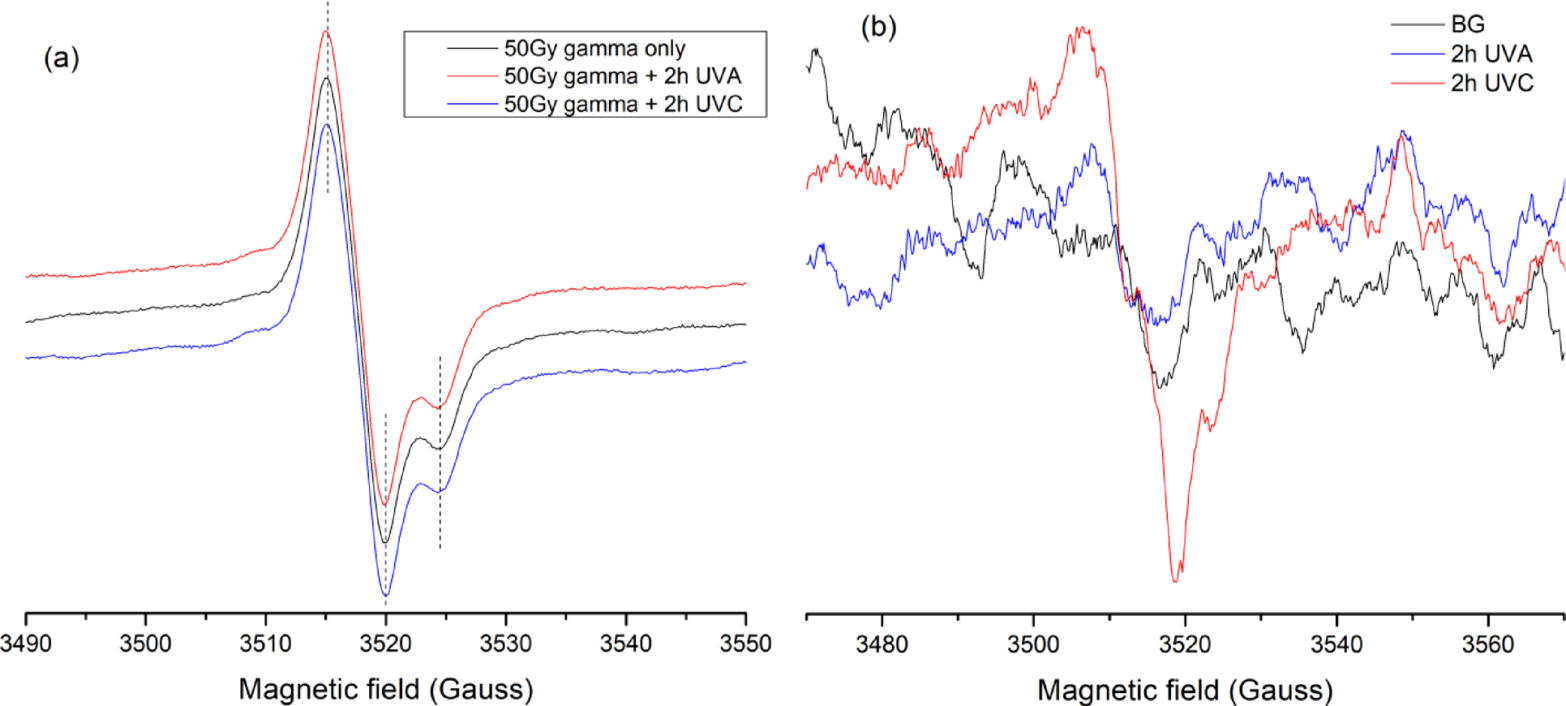
**a** The effect of UV light on EPR spectra of Oesh 2 days after exposure to 2 h of UVA and 2 h of UVC on 50 Gy of γ-irradiated egg shell. **b** The spectrum of exposed Ostrich egg shell to 2 h of both UVA and UVC light measured after 9 days of exposure compared to unirradiated one.

To investigate the dosimetric characteristics of Oesh for reliable retrospective dose assessment, the influence of non-ionizing radiation (UVA and UVC) on the EPR signal shape and intensity was examined for both unirradiated and gamma-irradiated samples, as illustrated in Fig. 8. EPR measurements were conducted on both gamma-irradiated and unirradiated egg shells two days following a 2 h UV exposure. Figure 8a shows that neither UVA nor UVC exposure produces an additional spectral component overlapping with the dosimetric peak observed in samples irradiated with 50 Gy of gamma rays. The RIS peak-to-peak intensity measured before and after UV exposure did not exhibit any statistically significant variation within experimental uncertainty, indicating that no measurable bleaching effect occurred under the present irradiation and exposure conditions. Furthermore, both UVA and UVC did not initiate a spectrum with the same shape as that observed in gamma irradiated egg shell (Fig. 8b). While the LIS recorded nine days after UVA exposure to 2 h was not accurately distinguishable from the background (BG) spectrum, the LIS of UVC was detectable. However, this UVC signal remained negligible compared to RIS from 50 Gy of gamma rays. The more prominent appearance of the LIS from UVC compared to UVA may be partially attributed to the UVC irradiance being four times higher than that of the UVA source.

These findings suggest that, under the applied conditions, UV exposure does not significantly affect the gamma-induced dosimetric signal. Nevertheless, the potential influence of UVC-induced signals may become relevant at very low gamma doses and warrants further dedicated investigation.

A comparison between the EPR dosimetric characteristics of Chicken egg shells reported in previous literature and the Ostrich egg shells investigated in this study is provided in Table 4.

**Table 4:** Comparative analysis of the retrospective dosimetric properties of Ostrich and Chicken egg shells.

Property	Chicken egg shell	Ostrich egg shell
Detection limit (MDD)	≈ 1–1.2 Gy^6,10^	≈ 0.2 Gy (D_DL_)
Linear dose range	3 Gy – 1 kGy^10^	0.2 Gy – 1 kGy
Fading behavior	≈ 55% after first 8 days, then stabilizes^10^	≈ 15% after first 7 days, then stabilizes
UV light resistance	Relatively UV light independent^6^	UVA independent on unirradiated, UVA and UVC independent on 50 Gy gamma.
Dominant EPR radicals	CO_3_^3−^, CO_3_^−^, and CO_2_^−^^7,10,26^	CO_3_^3−^, CO_3_^−^, and CO_2_^−^^8^

## Conclusions

The present study successfully investigated the EPR dosimetric characteristics of Ostrich egg shell, confirming its strong potential as an accurate and reliable retrospective dosimeter.

- Ostrich egg shell is a highly sensitive EPR dosimeter, achieving as low a detection limit as 0.21 Gy.
- The material exhibits wide-range linearity in its photon dose response from 0.3 Gy to 1 kGy, and its measurable range extends up to 50 kGy.
- The stability of the dosimetric signal is robust: it exhibits an initial fading but then stabilizes after 7 days at ambient temperature for the duration of the study (180 days).
- Under the present experimental conditions, UVA exposure did not induce a measurable light-induced signal, whereas UVC exposure produced a weak but detectable LIS. Although negligible compared to the RIS at moderate doses, this signal may become relevant in dose assessment at very low gamma doses and therefore warrants consideration.
- The time-dependent transformation of the radiation-induced radicals, quantified by the intensity ratio of the S_4_/(S_4_ + S_5_) peaks. This provides a unique method to accurately calculate the elapsed time after irradiation using an empirical third-order exponential decay model.
- The measured energy dependence factor agrees exactly with theoretical calculation for intermediate-energy photons (i.e., Cs-137 relative to Co-60) is approximately unity (1.01), confirming its efficacy and predictable response across common gamma energies.

Ultimately, the combination of high sensitivity, broad dose linearity, and robust temporal and thermal stability, alongside its resistance to UVA light, strongly supports the recommendation of using Ostrich egg shell material as a reliable and accurate for retrospective dosimetry and environmental radiation monitoring.

## Data Availability

Data sets generated during the current study are available from the corresponding author on reasonable request.

## Appendix

### A.1.1. Single hit (first order) saturation model

The single-hit saturation model assumes that each radiation-sensitive site requires a single energy deposition event to form a stable paramagnetic center. The dose–response relationship is expressed as:

10 $$I\left(D\right)={I}_{0}+A\left[1-exp\left(\frac{-D}{{D}_{0}}\right)\right]$$

where $I\left(D\right)$ is the measured EPR intensity at absorbed dose $D$, ${I}_{0}$​ represents the residual background signal, $A$ is the saturation amplitude corresponding to the maximum radical yield, and ${D}_{0}$​ is the characteristic saturation dose. Physically, ${D}_{0}$​ represents the dose at which approximately 63% of the available radiation-sensitive trapping sites associated with this dominant component are filled^39,40^.

### A.1.2. Two-component (multi-hit) saturation model

To account for more complex dose–response behavior, the signal may be described as the superposition of two independent first-order saturation processes:

11 $$I\left(D\right)={I}_{0}+{A}_{1}\left[1-exp\left(\frac{-D}{{D}_{01}}\right)\right]+{A}_{2}\left[1-exp\left(\frac{-D}{{D}_{02}}\right)\right]$$

Here, ${A}_{1}$​, ${D}_{01}$​ and ${A}_{2}$​, ${D}_{02}$ represent the amplitudes and characteristic saturation doses of two contributing components. This formulation does not imply that two distinct radical species are necessarily present; rather, it reflects the presence of multiple apparent saturation processes, which may arise from radicals formed at different trapping environments, defect sites, or microstructural regions within the material.

## References

[1] Aboelezz, E. & Pogue, B. W. Review of nanomaterial advances for ionizing radiation dosimetry. *Appl. Phys. Rev.*10.1063/5.0134982 (2023). 37304732 10.1063/5.0134982 PMC10249220

[2] Yang, Z., Vrielinck, H., Jacobsohn, L. G., Smet, P. F. & Poelman, D. Passive dosimeters for radiation dosimetry: materials, mechanisms, and applications. *Adv Funct. Mater* **34**, 2406186 (2024).

[3] Kalayci, T., Altug, D. T., Kinayturk, N. K. & Tunali, B. Characterization and potential usage of selected egg shell species. *Sci. Rep.* **15**, 1–20 (2025). 39979364 10.1038/s41598-025-87786-y PMC11842804

[4] Fattibene, P. et al. The 4th international comparison on EPR dosimetry with tooth enamel: Part 1: Report on the results. *Radiat. Meas.* **46**, 765–771 (2011).

[5] Hassan, G. M., Aboelezz, E., El-Khodary, A. & Eissa, H. M. Inter-comparison study between human and cow teeth enamel for low dose measurement using ESR. *Nucl. Instrum. Methods Phys. Res. B* **268**, 2329–2336 (2010).

[6] Engin, B. & Demirtaş, H. The use of ESR spectroscopy for the investigation of dosimetric properties of egg shells. *Radiat. Phys. Chem.* **71**, 1113–1123 (2004).

[7] Koksal, F., Demir, D., Koseoglu, R., Birey, M. & Koroglu, A. Applied magnetic resonance EPR of γ-irradiation-induced free radicals in chicken, duck, and quail egg shells. *Appl Magn. Reson* **29**, 205-210 (2005).

[8] Bhatti, I. A., Akram, K., Ahn, J. J. & Kwon, J. H. Electron spin resonance analysis of radiation-induced free radicals in shells and membranes of different poultry eggs. *Food Anal. Methods* **6**, 265–269 (2013).

[9] Vazirov, R. A., Sokovnin, S. Y., Agdantseva, E. N. & Tsmokalyuk, A. N. Investigation of radiation-induced electron paramagnetic resonance signal of an egg shell after electron beam irradiation. *Radiat. Phys. Chem.*10.1016/j.radphyschem.2021.109882 (2022).

[10] Panda, M. et al. Chicken egg shells as unconventional ESR dosimeters: Insight into the influence of particle size and fading characteristic. *J. Radioanal. Nucl. Chem.* **334**, 1919–1929 (2025).

[11] Regulla, D. F., Göksu, H. Y., Vogenauer, A. & Wieser, A. Retrospective dosimetry based on egg shells. *Appl. Radiat. Isot.* **45**, 371–373 (1994).

[12] Kai, A., Miki, T. & Ikeya, M. ESR dating of teeth, bones and egg shells excavated at a Paleolithic site of Douara Cave, Syria. *Quat. Sci. Rev.* **7**, 503–507 (1988).

[13] Mikhailov, K. E. & Zelenkov, N. The late Cenozoic history of the ostriches (Aves: Struthionidae), as revealed by fossil egg shell and bone remains. *Earth. Sci. Rev.* **208**, 103270 (2020).

[14] Miller, J. M. & Wang, Y. V. Ostrich egg shell beads reveal 50,000-year-old social network in Africa. *Nature* **601**, 234–239 (2022). 34931044 10.1038/s41586-021-04227-2 PMC8755535

[15] Roberts, P. et al. Climate, environment and early human innovation: Stable isotope and faunal proxy evidence from archaeological sites (98-59 ka) in the southern Cape, South Africa. *PLoS One* **11(7)**: e0157408 (2016). 10.1371/journal.pone.0157408 PMC4934875 27383620

[16] Christensen, V. L., Davis, G. S. & Lucore, L. A. Egg shell conductance and other functional qualities of ostrich eggs. *Poult. Sci.* **75**, 1404–1410 (1996). 8933594 10.3382/ps.0751404

[17] Chiang, P. L. et al. Elastic moduli of avian egg shell. *Biology (Basel)* **10(10)**, 989 (2021). 10.3390/biology10100989 PMC8533214 34681088

[18] Pérez-Huerta, A., Brugal, J. P., Salomé, M., Schmitt, C. N. Z. & Dauphin, Y. Integrated information on the structure and composition of the ostrich egg shell (*Struthio camelus*). *Minerals*10.3390/min13040481 (2023).

[19] Marciniak, A. et al. Categorization of screen glasses of mobile devices with respect to their EPR spectral properties and potential applicability for use in retrospective dosimetry. *Front. Public Health*10.3389/fpubh.2025.1659601 (2025). 40994757 10.3389/fpubh.2025.1659601 PMC12454032

[20] Aboelezz, E. & Sharaf, M. A. Effects of selected physical parameters on the gamma-ray-induced EPR signal of glycine dosimeter. *Eur. Phys. J. Plus*10.1140/epjp/s13360-024-05876-8 (2024).

[21] Aboelezz, E. & Hassan, G. M. Resolving the limitations of using glycine as EPR dosimeter in the intermediate level of gamma dose. *Radiat. Phys. Chem.* **145**, 5–10 (2018).

[22] Aboelezz, E., De Angelis, C. & Fattibene, P. A study on energy dependence of nano barium sulfate powder using the EPR technique in photon, electron and proton beams. *Measurement* **175**, 109108 (2021).

[23] Seltzer, S. M. Calculation of photon mass energy-transfer and mass energy-absorption coefficients. *Radiat. Res.* **136**, 147–170 (1993). 8248472

[24] Koşarsoy Ağçeli, G. Development of ostrich egg shell and nano-levan-based edible biopolymer composite films: Characterization and bioactivity. *Polym. Bull.* **79**, 11201–11215 (2022).

[25] Joint Committee for Guides in Metrology. JCGM 100:2008. Evaluation of Measurement Data—Guide to the Expression of Uncertainty in Measurement (2008).

[26] Da Costa, Z. M. et al. A study based on ESR, XRD and SEM of signal induced by gamma irradiation in egg shell. *Radiat. Meas.* **42**, 1233–1236 (2007).

[27] Wang, S., Jia, Z., Gao, H., Xue, D. & Pan, B. Impact of microwave power on equivalent dose (De) evaluation in ESR dating. *Radiat. Meas.* **177**, 107231 (2024).

[28] Hassan, G. M. & Sharaf, M. A. ESR dosimetric properties of some biomineral materials. *Appl. Radiat. Isot.* **62**, 375–381 (2005). 15607478 10.1016/j.apradiso.2004.08.013

[29] Iwasaki, M., Miyazawa, C., Uesawa, T. & Niwa, K. Effect of sample grain size on the CO33- signal intensity in ESR dosimetry of human tooth enamel. *Radioisotopes* **42**, 470–473 (1993).

[30] Hayes, R. B., Haskell, E. H., Romanyukha, A. A. & Kenner, G. H. Technique for increasing reproducibility in EPR dosimetry of tooth enamel. *Meas. Sci. Technol.* **9**, 1994–2006 (1998).

[31] Kumar, P. et al. Site-Selective Zn^2+^ substitution induced magnetic phase transition from ferrimagnetism to superparamagnetism in N,N,N-trimethylhexadecan-1-aminium bromide stabilized Co–Zn ferrite nanoparticles. *J. Alloys Compd.* **1050**, 185745 (2026).

[32] Fattibene, P. & Callens, F. EPR dosimetry with tooth enamel: A review. *Appl. Radiat. Isot.* **68**, 2033–2116 (2010). 20599388 10.1016/j.apradiso.2010.05.016

[33] Paksu, U. & Engin, B. Electron spin resonance (ESR) study on gamma irradiated some modern paper samples. *Radiat. Meas.*10.1016/j.radmeas.2022.106849 (2022).

[34] Currie, L. A. Nomenclature in evaluation of analytical methods including detection and quantification capabilities (IUPAC Recommendations 1995). *Pure Appl. Chem.* **67**, 1699–1723 (1995).

[35] International Organization for Standardization. ISO 11929:2019. Determination of the Characteristic Limits (Decision Threshold, Detection Limit and Limits of the Coverage Interval) for Measurements of Ionizing Radiation—Fundamentals and Application (2019).

[36] Aboelezz, E., Hassan, G. M. & Sharaf, M. A. El-Khodary, A. EPR dosimetric properties of nano-barium sulfate. *Radiat. Phys. Chem.* **106**, 385–393 (2015).

[37] Burlin, T. E. A general theory of cavity ionisation. *Br. J. Radiol.*10.1259/0007-1285-39-466-727 (1966). 5927191 10.1259/0007-1285-39-466-727

[38] Attix, F. H. *Introduction to Radiological Physics and Radiation Dosimetry* (Wiley, 1986).

[39] Barabas, M., Mudelsee, M., Walther, R. & Mangini, A. Dose-response and thermal behaviour of the ESR signal at g = 2.0006 in carbonates. *Quat. Sci. Rev.* **11**, 173–179 (1992).

[40] Ciesielski, B. Combined effects of high doses and temperature on radiation-induced radicals and their relative contributions to EPR signal in gamma-irradiated alanine. *Radiat. Prot. Dosim.* **120**, 184–190 (2006). 10.1093/rpd/nci503 16565208

